# Prenatal Adversity Alters the Epigenetic Profile of the Prefrontal Cortex: Sexually Dimorphic Effects of Prenatal Alcohol Exposure and Food-Related Stress

**DOI:** 10.3390/genes12111773

**Published:** 2021-11-09

**Authors:** Alexandre A. Lussier, Tamara S. Bodnar, Michelle Moksa, Martin Hirst, Michael S. Kobor, Joanne Weinberg

**Affiliations:** 1 Psychiatric and Neurodevelopmental Genetics Unit, Center for Genomic Medicine, Massachusetts General Hospital, Boston, MA 02114, USA; 2 Department of Psychiatry, Harvard Medical School, Boston, MA 02115, USA; 3 Stanley Center for Psychiatric Research, Broad Institute of MIT and Harvard, Cambridge, MA 02142, USA; 4 Department of Cellular and Physiological Sciences, Faculty of Medicine, Life Sciences Institute, University of British Columbia, Vancouver, BC V6T 1Z3, Canada; tamara.bodnar@ubc.ca; 5 Department of Microbiology and Immunology, Michael Smith Laboratories, University of British Columbia, Vancouver, BC V6T 1Z4, Canada; mmoksa@mail.ubc.ca (M.M.); hirstm@mail.ubc.ca (M.H.); 6 Canada’s Michael Smith Genome Sciences Centre, BC Cancer, Vancouver, BC V5Z 4S6, Canada; 7 BC Children’s Hospital Research Institute, Vancouver, BC V5Z 4H4, Canada; msk@bcchr.ca; 8 Department of Medical Genetics, University of British Columbia, Vancouver, BC V5Z 4H4, Canada; 9 Centre for Molecular Medicine and Therapeutics, Vancouver, BC V5Z 4H4, Canada; 10 Program in Child and Brain Development, CIFAR, MaRS Centre, West Tower, 661 University Ave., Suite 505, Toronto, ON M5G 1M1, Canada

**Keywords:** fetal alcohol spectrum disorder, DNA methylation, sex differences, autism, development, brain, prefrontal cortex, epigenetics

## Abstract

Prenatal adversity or stress can have long-term consequences on developmental trajectories and health outcomes. Although the biological mechanisms underlying these effects are poorly understood, epigenetic modifications, such as DNA methylation, have the potential to link early-life environments to alterations in physiological systems, with long-term functional implications. We investigated the consequences of two prenatal insults, prenatal alcohol exposure (PAE) and food-related stress, on DNA methylation profiles of the rat brain during early development. As these insults can have sex-specific effects on biological outcomes, we analyzed epigenome-wide DNA methylation patterns in prefrontal cortex, a key brain region involved in cognition, executive function, and behavior, of both males and females. We found sex-dependent and sex-concordant influences of these insults on epigenetic patterns. These alterations occurred in genes and pathways related to brain development and immune function, suggesting that PAE and food-related stress may reprogram neurobiological/physiological systems partly through central epigenetic changes, and may do so in a sex-dependent manner. Such epigenetic changes may reflect the sex-specific effects of prenatal insults on long-term functional and health outcomes and have important implications for understanding possible mechanisms underlying fetal alcohol spectrum disorder and other neurodevelopmental disorders.

## 1. Introduction

Neurodevelopmental and psychiatric disorders may be partly rooted in early-life environments, which can have profound influences on cognitive, neurobiological, and physiological outcomes. For instance, autism spectrum disorder (ASD) is influenced by both genetic mechanisms and environmental factors, such as prenatal maternal stress [1,2], medication use [3], and maternal immune dysfunction [2], among others (reviewed in [4]). By contrast, fetal alcohol spectrum disorder (FASD) has more clearly defined roots, with prenatal alcohol exposure (PAE) being the key etiological factor, although, as in ASD, environmental factors such as maternal nutrition, health, and stress can significantly influence outcome. Although the mechanisms linking environmental exposures to neurodevelopmental outcomes are not fully understood, one prevailing hypothesis is that the effects of early-life challenges become biologically embedded through epigenetic mechanisms, such as histone modifications, non-coding RNA expression, and DNA methylation (DNAm) [5,6]. The latter is the most commonly studied epigenetic modification and involves the addition of a methyl residue to the cytosines. Importantly, DNAm is relatively stable over time and may capture the effects of environmental exposures to modulate long-term gene expression, functional outcomes, and health [7].

FASD describes the wide range of cognitive, behavioral, adaptive, and physiological alterations that occur following PAE [8]. In addition to its direct teratogenic effects, PAE can program or sensitize key neurobiological and physiological systems, thus increasing later life vulnerabilities to adverse functional and health outcomes. Systems involved in regulation of the stress response, particularly, the hypothalamic-pituitary-adrenal (HPA) and immune systems, are highly susceptible to programming and altered by PAE. Indeed, in both animal model and clinical studies, PAE resulted in HPA dysregulation, including hyperresponsiveness to stressors [9], alterations in diurnal HPA regulation [10], and increased physical and mental health problems, including metabolic disorders [11], depression, and anxiety [12], and deficits in immune system activity and regulation [13]. As early life stress or adversity can result in some adverse outcomes parallel to those of PAE in adulthood [14,15], it is in this context that PAE can be considered a type of prenatal stressor.

Of particular relevance to the current study is the issue of sex differences in the adverse effects of PAE. Until recently, most studies utilizing animal models often excluded or failed to analyze data from females. Nevertheless, of those studies that probed for sex-specific changes, differential effects of PAE on males and females were reported in both rodent and primate models, including differences in hippocampal microglia and cytokine expression [16], hypothalamic-pituitary-adrenal (HPA) activity and regulation [9], dopaminergic regulation [17], immune responses [18], social behavior [19,20,21], and depressive- and anxiety-like behaviors [12,22,23,24]. By contrast, clinical research in the FASD field has typically included children of both sexes, and sex differences in prevalence of FASD, brain maturation, cognitive function, and mental health, among other outcomes, have been reported [25,26,27,28,29]. Although the biological mechanism that mediate altered developmental outcomes following PAE are not fully understood, several studies have revealed broad impacts of PAE on epigenetic patterns in the brain [30]. While the majority of studies limit their analyses to either male or female subjects, recent evidence from candidate gene analyses suggests that PAE may have sexually dimorphic effects on epigenetic profiles [31,32,33]. Emerging evidence from human populations also suggests that biological sex influences autosomal DNAm patterns, further highlighting the importance of considering sex in epigenome-wide analyses [34]. However, no studies have investigated whether there is a genome-wide and sex-specific impact of PAE on the epigenome of cell types that contribute to the brain, limiting our ability to identify the molecular mechanisms that may drive sexual dimorphisms associated with PAE, as well as their overlaps with other neurodevelopmental disorders such as ASD.

Of note, our animal model of PAE and that of many others includes not only an ad libitum-fed control diet group but also a secondary control, the pair-fed (PF) group. Pair-feeding is a standard procedure to control for the reduced food intake of animals consuming alcohol; PF animals get a reduced ration, matched to that of a PAE partner, and thus less than what would be consumed in a diet without alcohol. This results in hunger, abnormal feeding patterns (consuming most of the ration within a few hours of feeding and remaining food deprived for the remainder of the 24-h period), and mild stress. A treatment in itself, pair-feeding can reprogram offspring behavior and physiological functions, such as alterations to stress system regulation [35,36], reproductive development and function [37,38], immune system development [39], as well as depressive- and anxiety-like behavior [24], among other outcomes. Studies on food scarcity or restriction in human populations have revealed parallel insights, showing that alterations to food access can have marked effects on the programming of physiological systems [40,41], particularly if deficiencies occur during critical or sensitive periods of brain or organ development. To this end, several studies have investigated the effects of severe food scarcity on the developing fetus, particularly in the context of the Dutch Famine or Hunger Winter, identifying sexually-dimorphic effects on both physiological outcomes, such as metabolic disorders and brain function [42], as well as DNAm patterns linked to growth and metabolism [43] that persist across the life course [44] and that are sex-specific [45]. The present study is one of the first to investigate the impact of food-related stress/food restriction at the epigenome-wide level in the brain, with the aim of increasing our understanding of the long-term effects of food-related stress on developmental processes.

The goals of the present study were to (1) identify sex-specific alterations to DNAm in response to prenatal adversity; (2) identify sex-concordant alterations to DNAm resulting from prenatal adversity; and (3) assess the shared etiology of genes influenced by PAE and food-related stress. We utilized a well-established rat model of PAE to examine the impact of two early-life exposures—PAE and food-related stress—on genome-wide DNAm patterns of the prefrontal cortex (PFC). The PFC plays key roles in many important higher order functions including cognition/executive function, working memory, decision making, planning and behavioral flexibility, regulation of affective behavior, and social reasoning [46,47]. Importantly, the PFC is also responsive to stressors and glucocorticoid levels, modulating the behavioral and physiological responses to stress through regulation of the paraventricular nucleus of the hypothalamus, which, in turn, controls both autonomic and neuroendocrine functions [48,49]. Furthermore, we investigated the potential relevance of this impact for understanding neurodevelopmental disorders beyond FASD, specifically, ASD. We focused on ASD due to its phenotypic overlaps with FASD despite differences in core phenotypic characteristics, as well as reported co-morbidity with FASD [50,51,52], which point to potential shared etiologies that may be further uncovered in these analyses. Importantly, our results provide insight into the biological pathways that influence the sexual dimorphic outcomes resulting from prenatal insults, such as alcohol exposure, stress, and food deprivation, while highlighting potential pathways driving the phenotypic overlaps between FASD and ASD.

## 2. Materials & Methods

### 2.1. Prenatal Treatments

All animal protocols were approved by the University of British Columbia Animal Care Committee and are consistent with the NIH Guide for the Care and Use of Laboratory Animals (National Research Council 2011). Details of the procedures for breeding, feeding, and handling have been published previously [35]. Briefly, nulliparous Sprague-Dawley females (*n* = 39) were pair-housed with a male, and vaginal lavage samples were collected daily for estrous cycle staging and to check for the presence of sperm, indicating gestation day 1 (GD1). Pregnant dams were then singly housed and assigned to one of three prenatal treatment groups: Prenatal alcohol exposure (PAE)—ad libitum access to liquid ethanol diet, 36% ethanol-derived calories, 6.37% v/v, *n* = 13; Pair-fed (PF)—liquid-control diet, maltose-dextrin isocalorically substituted for ethanol, in the amount consumed by a PAE partner, g/kg body weight/GD), *n* = 14—this represents a restricted feeding condition; or Control (CON)—pelleted version of the liquid control diet, ad libitum, *n* = 12. All animals had ad libitum access to water. Experimental diets (Weinberg/Kiever Liquid Ethanol Diet #710324, Weinberg/Kiever Liquid Control Diet #710109, and Pelleted Control Diet #102698, Dyets Inc., Bethlehem, PA, USA) were provided from gestation days 1–21, and then replaced with standard laboratory chow (19% protein). Litters were weighed and culled at birth to 6 males and 6 females, when possible. These litters are the same as those used as in our previous study of hypothalamic and white blood cell samples [53].

### 2.2. Sample Collection and DNA Extraction

**Figure 1:**
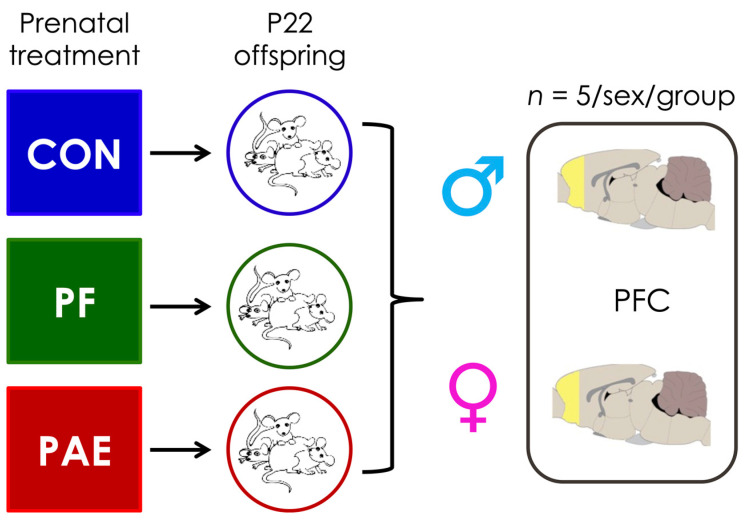
Study design. The prefrontal cortex (PFC) was collected from 5 female and 5 male animals for each of the three prenatal treatment groups (control [CON], pair-fed [PF], prenatal alcohol exposed [PAE]) on postnatal day 22 (P22).

On postnatal day 22, we selected female and male offspring from this large breeding as subjects for the current study (*n* = 5/group/sex, no more than 1 male and 1 female/litter to control for litter effects). Animals were decapitated, and brains were removed and weighed; the prefrontal cortex (PFC) was then quickly dissected and frozen on dry ice in RNA later (Qiagen, Hilden, Germany); Figure 1. All tissue collected was left at 4 °C for 1 day and then frozen at −80 °C until DNA extraction. Total DNA was extracted from the PFC using the RNA/DNA extraction kit (Qiagen, Hildren, Germany). Cells were mechanically lysed using the Omni Bead Ruptor Elite (Omni International, Kennesaw, GA, USA). DNA concentration was assessed using Qubit Fluorometric Quantitation (Life Technologies, Carlsbad, CA, USA).

### 2.3. Methylated DNA Immunoprecipitation and Next-Generation Sequencing

Methylated DNA immunoprecipitation followed by next-generation sequencing (meDIP-seq) procedures were performed as previously described [53,54]. To summarize briefly, we performed immunoprecipitation of 5-methylcytosine of adapted gDNA to obtain an enriched fraction of methylated DNA fragments. These fragments were then amplified by PCR, pooled, and sequenced using the Illumina HiSeq 2000 at Canada’s Michael Smith Genome Science Centre (Vancouver, BC, Canada) using paired-end sequencing. The resulting fastq files were split by index, assessed for quality, and paired for downstream analysis.

### 2.4. Bioinformatic Analyses

#### 2.4.1. Next-Generation Sequencing Quality Control

Fastq files were aligned to the most current rat genome (Rn6, July 2014) using BWA to obtain .bam files [55]. Bam files were sorted and filtered using samtools to remove duplicate reads, unpaired reads, and reads with a minimum mapping quality score below 10.

#### 2.4.2. Peakset Generation

Model-based analysis of ChIP-seq (MACS2; version 2.1.2) was used to identify enriched regions of DNAm across the genome [56] as previously described [53]. Briefly, the peak regions were identified using the ‘callpeaks’ function on paired end bam files with no control input and the following options: –f BAMPE –m 5 50 –bw 300 –g 2.9 × 10^9^ –q 0.05. Each sample was modeled individually, generating 30 total peaksets. Peaksets were imported into R using the DiffBind package [57,58] and combined into common regions using the dba.count function in DiffBind. Specifically, this set removed peaks found in fewer than 3 samples and calculated the total number of reads within each peak/sample. Sex chromosomes were removed from the final dataset, which contained 358,773 meDIP-seq peaks.

#### 2.4.3. Data Preprocessing and Normalization

Reads within each peak were converted to reads per kilobase per million (RPKM). Variation associated with batch effects were corrected using the ComBat method from the sva package (version 3.32.1), protecting the effects of prenatal treatment group and sex.

### 2.5. Differentially Methylated Region (DMR) Identification

Linear modeling was performed using edgeR (version 3.26.8) to identify DMRs that were: (1) sex-concordant (~group + sex); (2) female-specific (~group; females only); or (3) male-specific (~group; males only). *p*-values were corrected for multiple-testing using the Benjamini-Hochberg method [59]. Significant DMRS at a false discovery rate (FDR) < 0.05 were obtained for the following contrasts: PAEvCON, PAEvPF, and PFvCON. The final PAE-specific DMRs were significant in both PAEvCON and PAEvPF but not the PFvCON contrasts. The final PF-specific DMRs were statistically significant in both PFvCON and PAEvPF but not the PAEvCON contrasts. The final shared DMRs between PAE and PF were statistically significant in both the PAEvCON and PFvCON contrasts.

### 2.6. Genomic Enrichment

A custom annotation was built for the peakset using the UCSC genome browser gene annotations. Briefly, genomic coordinates of all CpG islands, exons, introns, promoters (TSS-200 bp and TSS -1500 bp), 3′ untranslated regions (UTR), 5′ UTRs for the rn6 genome were obtained as bed files from the table browser. These were intersected with the meDIP-seq peaks uusing the intersectBed function from bedtools. The overlaps were concatenated into a single annotation set in R, where individuals peaks contained information for each potential genomic feature. Of note, regions spanning both introns and exons were deemed intron/exons boundaries and a given DMR could span multiple genomic features. *p*-values for genomic feature enrichment analyses were calculated by computing background levels of genomic features on 10,000 random subsets of DMRs, using the same number of PAE-specific, PF-specific, or shared DMRs.

### 2.7. Gene Ontology Analyses

The gene-score resampling (GSR) tool of ErmineR (version 1.0.1.9) was used to identify gene function enrichment in the differentially methylated genes including the Gene Ontology (GO) annotations molecular function, biological process, and cellular component [60,61]. The ErmineR gene score resampling (GSR) tool was set with the following parameters: max gene set size = 2000; min gene set size = 2; iterations = 10,000. Significant associations (FDR < 0.05 and corrected multifunctionality *p*-value < 0.05) were obtained for the following contrasts: PAEvCON, PAEvPF, and PFvCON. The final PAE-specific GO terms were statistically significant in both the PAEvCON and PAEvPF, but not the PFvCON contrasts. The final PF-specific GO terms were statistically significant in both the PFvCON and PAEvPF, but not the PAEvCON contrasts. The final shared GO terms between PAE and PF were significant in both the PAEvCON and PFvCON contrasts.

### 2.8. Chi-Squared Tests of the Direction of DMRs

We determined whether the proportion of down-methylated or up-methylated DMRs was significantly different from the expected proportion of 50% using the chisq.test function in R with simulated *p*-values.

## 3. Results

We performed three main sets of analyses, first focusing on identifying PAE-specific DMRs, followed by DMRs linked to food-related stress (PF group), and DMRs shared between PAE and PF. Within these main analyses, we further explored sex-concordant and sex-specific alterations to DNAm patterns within the PFC. We note that any DMRs that overlapped between sex-specific and sex-concordant analyses were assigned to the sex-specific analysis, as they were likely driven by that sex in the sex-concordant analysis. Finally, we performed a qualitative analysis of genes overlapping with autism spectrum disorder to determine if there are common pathways underlying FASD and ASD.

### 3.1. PAE Resulted in Sex-Concordant Alterations to DNAm Patterns

**Figure 2:**
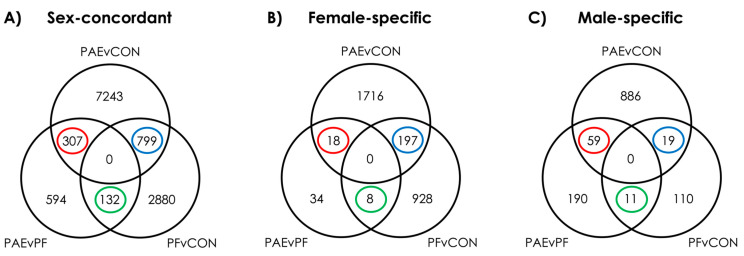
Differentially methylated regions were identified for sex-concordant and sex-specific analyses at a false-discovery rate < 0.05. (**A**) Analysis of sex concordant effects (DNAm~group + sex) revealed 307 PAE-specific DMRs (red), 132 PF-specific DMRs (green), and 799 DMRs shared between PAE and PF (purple). (**B**) Analysis of female-specific DMRs (DNA~group; females only) revealed 18 PAE-specific, 8 PF-specific DMRs, and 197 DMRs shared between PAE and PF (purple). (**C**) Analysis of male-specific DMRs (DNA~group; males only) revealed 59 PAE-specific, 11 PF-specific DMRs, and 19 DMRs shared between PAE and PF (blue). Diagram circles represent the three contrasts performed for each analysis.

**Figure 3:**
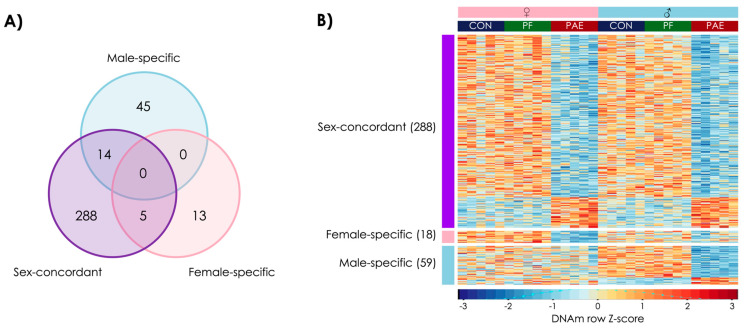
PAE induced sex-concordant and sex-specific alterations to DNA methylation patterns. (**A**) Venn diagram showing the overlap among the three sets of PAE-specific differentially methylated regions (DMRs) at a false-discovery rate < 0.05. 307 DMRs were identified in the analysis of both sexes together, with 5 driven primarily by females and 14 driven primarily by males. As such, 18 DMRs were categorized as female-specific and 59 were categorized as male-specific. (**B**) Heatmap of the DMRs, where each row is a DMRs, scaled to Z-score of DNAm, and each column is a different animal. Most DMRs showed a decrease in the PAE (red) compared to the CON (blue) and PF (green) animals.

To assess sex-concordant alterations to DNAm patterns following PAE, we performed linear modeling with both sexes included, utilizing a model that accounted for sex. Contrast analyses to identify PAE-specific alterations successfully identified 307 PAE-specific DMRs at an FDR < 0.05 (Figure 2). However, 14 of these overlapped with the male-specific DMRs and 5 overlapped with the females-specific DMRs (Figure 3A). As such, we found 288 sex-concordant DMRs that were consistent across both sexes and showed consistently different DNAm levels in PAE compared to CON and PF animals (Figure 3B; Supplementary Table S1).

Of these, 46 were up-methylated and 242 were down-methylated in PAE versus both CON and PF groups (χ^2^ = 75.4; *p* = 0.0005), with sizes ranging from 271 to 1894 bp (median = 465 bp). Furthermore, 193 of the DMRs showed at least 1.5-fold change in DNAm levels in PAE versus both CON and PF animals (Supplementary Table S1), suggesting that PAE could induce robust sex-concordant alterations to DNAm patterns.

Overall, 119 DMRs were located in genes, several of which were involved in potassium channel activity (*Kcnn1*, *Kcnn1*, *Kcnh5*, *Kcnip1*, *Kcnq1*) and ion signaling (*Grik1*, *Camk2d*, *Itpr2*, *Slc12a8*). Of note, five genes, *Camta1*, *Cpne4*, *Ephb1*, *Magi1*, and *Tmem178b*, had multiple DMRs (Supplementary Table S1). The majority of DMRs were found in intergenic regions, but also showed lower enrichment in these regions than by random chance (*p* = 0.0018). By contrast, DMRs showed increased enrichment in exons (*p* = 0.026) and introns (*p* = 0.0018), which frequently spanned intron/exon boundaries.

Using gene-score enrichment, we identified 15 PAE-specific biological processes that were enriched in a sex-concordant manner. These included pathways involved in central nervous system development, metabolic processes, and the inflammatory response (Supplementary Table S2).

### 3.2. PAE Resulted in Sex-Specific Alterations to DNAm Patterns

Moving beyond sex-concordant alterations, we performed a sex-stratified analyses using linear modeling to identify sex-specific alterations following PAE. Contrast analyses revealed PAE-specific alterations at 18 DMRs in females and 59 DMRs in males at an FDR < 0.05) (Figure 3A; Supplementary Table S1).

All 18 female-specific DMRs showed decreased DNAm in PAE compared to CON and PF animals (χ^2^ = 12; *p* = 0.002), which ranged from 279 to 607 bp in length (median = 377 bp). Of these, 7 DMRs were located in genes. Female-specific DMRs did not show any differences in genomic location enrichment compared to the background of the dataset. Five PAE-specific biological processes were identified, including those involved in acetylcholine and angiotensin receptor functions (Supplementary Table S2).

In males, 48 DMRs showed decreased DNAm and 11 showed increased DNAm in PAE compared to CON and PF animals (χ^2^ = 12.3; *p* = 0.001). These male-specific DMRs ranged from 291 to 3300 bp (median = 417 bp), and 15 DMRs were located in genes. Again, no significant enrichment for genomic features was detected. Six PAE-specific biological processes included those involved in the regulation of hormone metabolism and other metabolic processes (Supplementary Table S2).

### 3.3. Prenatal Food-Related Stress Had Both Sex-Concordant and Sex-Specific Effects

**Figure 4:**
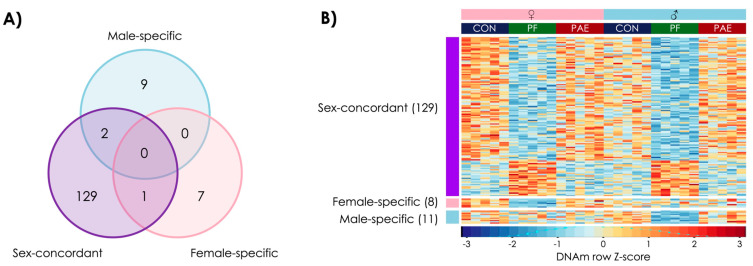
Food-related stress caused sex-concordant and sex-specific alterations to DNA methylation. (**A**) Venn diagram showing the overlap among the three sets of PF-specific differentially methylated regions (DMRs). 132 DMRs were identified in the analysis of both sexes together, with 1 driven primarily by females and 2 driven primarily by males. As such, 8 DMRs were categorized as female-specific and 11 were categorized as male-specific. (**B**) Heatmap of the DMRs, where each row is a DMRs, scaled to Z-score of DNAm, and each column is a different animal. Most DMRs showed a decrease in the PF (green) compared to the CON (blue) and PAE (red) animals.

Next, we investigated the effects of pair-feeding, a restricted feeding paradigm that in itself induces prenatal stress related to hunger and disrupted feeding patterns. As noted, this treatment may capture some elements of food insecurity or scarcity on DNAm patterns of the PFC. Using parallel approaches to the PAE analyses, we identified 129 sex-concordant, 8 female-specific, and 11 male-specific DMRs that were driven by pair-feeding effects (Figure 4A; Supplementary Table S3).

Of the 129 sex-concordant DMRs, 100 showed decreased DNAm and 29 showed increased DNAm in PF compared to CON and PAE animals (χ^2^ = 20.6; *p* = 0.0005) (Figure 4B). 39 DMRs were located in genes, with most again being located in introns or intergenic regions. Of note, *Adarb2*, *Dgki*, and *Gpc5* contained two DMRs each, and perhaps most interestingly, one of the sex-concordant DMRs was located in an intronic region of *Nr3c1*, the gene coding for the glucocorticoid receptor (GR). We also identified 22 PF-specific biological processes from the sex-concordant analysis, of which several were involved in metabolic processes (Supplementary Table S4).

Similar to previous analyses that found a general decrease in DNAm from prenatal treatments, every sex-specific DMR identified here showed decreased DNAm in PF animals, suggesting that pair-feeding or food-related stress may also have generally inhibitory effects on DNAm patterns. Although few DMRs were located in genes (3/8 for females and 2/11 for males), we identified several PF-specific biological pathways that displayed sex-specific effects (Supplementary Table S4). In females, we identified 33 pathways that were involved in endocrine, immune, and metabolic processes, while in males, we identified 25 pathways, which were mainly involved in metabolic processes. Overall, these results suggest that the stress induced by pair-feeding is a distinct physiological stressor, which has broad and sexually-dimorphic effects on the regulatory mechanisms of the brain.

### 3.4. PAE and PF Animals Shared Some Sex-Concordant and Sex-Specific Effects on DNAm Patterns

**Figure 5:**
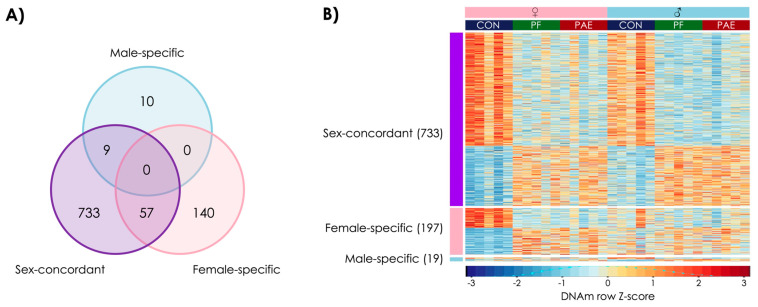
PAE and food-related stress shared sex-concordant and sex-specific DNA methylation changes. (**A**) Venn diagram showing the overlap among the three sets of differentially methylated regions (DMRs) shared between PAE and PF animals. 799 DMRs were identified in the analysis of both sexes together, with 57 driven primarily by females and 9 driven primarily by males. As such, 197 DMRs were categorized as female-specific and 19 were categorized as male-specific. (**B**) Heatmap of the DMRs, where each row is a DMRs, scaled to Z-score of DNAm, and each column is a different animal. Most DMRs showed a decrease in the PF (green) and PAE (red) compared to the CON (blue) animals.

Given that prenatal alcohol exposure and pair-feeding may share some common pathways in the reprogramming of biological systems, we also investigated DMRs shared between these two prenatal exposures. Here, we found 733 sex-concordant, 197 female-specific, and 19 male-specific DMRs that were influenced by both PAE and PF (Figure 5A; Supplementary Table S5).

Of the 733 sex-concordant, shared DMRs, 479 showed decreased DNAm and 254 showed increased DNAm in PAE and PF compared to CON animals (χ^2^ = 270; *p* = 0.0005) (Figure 4B). Of these, 309 were located in genes, including the transcription start site of Drd4, the dopamine D4 receptor gene, which has previously been associated with PAE [62,63,64]. We also identified 33 PAE and PF-shared biological pathways from the sex-concordant analysis, of which several were involved in metabolic processes and hormone regulation (Supplementary Table S6).

In contrast to the PAE- and PF-specific DMRs, 58% of shared DMRs in females showed an increase in DNAm in PAE and PF animals compared to CON (114 of 197 DMRs; χ^2^ = 3.1; *p* = 0.08), whereas in males, marginally more DMRs showed lower DNAm in PAE and PF animals compared to CON (10 of 19 DMRs; χ^2^ = 0; *p* = 1). Here, we identified 26 biological pathways that were enriched in females, including those involved in cellular stress and metabolism, and 10 biological pathways enriched in males, which were mainly involved in metabolic processes. These findings suggest that PAE and restricted feeding, both of which act in many respects as prenatal stressors, may influence some common biological pathways, which may explain some of the occasional overlap between their resulting phenotypes.

### 3.5. PAE-Specific and Shared DMRs Overlapped with Genes Linked to Autism Spectrum Disorder

Finally, we qualitatively assessed whether there were any overlaps of DMRs with genes previously implicated in ASD from genome-wide association studies (GWAS) [65] and epigenome-wide association studies (EWAS) on peripheral [66,67,68] or central tissues [69,70,71] (Table 1).

Comparing results from the most recent GWAS of ASD [65], we found one overlap with PAE-specific DMRs (NEGR1) and one overlap with shared DMRs (MMS22L). By contrast, we did not find any overlaps for PAE, PF, or shared DMRs with DNAm signatures of ASD in blood from EWAS studies in human populations [66,67]. However, we found one overlap between female-specific shared DMRs and a study of buccal epithelial cells from ASD cases (NRG2) [68]. Moreover, when we compared our current findings to a recent study of DNAm patterns in the PFC of individuals with ASD [70], we found one overlap with PAE-specific DMRs (CDH13) and one overlap with shared DMRs (PRKAR1B). Importantly, CDH13 was one of the few genes with multiple DMRs; in this instance, it contained two distinct DMRs that were identified in the male-specific and sex-concordant analyses. Findings from a cross-cortex analysis of ASD in the same study [70] also showed some overlaps with PAE-specific (GRIK1) and shared (FRMD4A) DMRs. Finally, we found another overlap between shared DMRs (NEDD4L) and an independent study of DNAm in nuclei isolated from the frontal cortex of men with ASD [69].

Of note, almost every overlapping gene was identified in the sex-concordant analyses, with the exception of NRG2 and CDH13, as noted above. These findings suggest that the shared pathways between autism spectrum disorder and early life stressors may be agnostic to the effects of sex. Furthermore, we found no overlaps between PF-associated DMRs and ASD genes identified at either the genetic or epigenetic level, suggesting potentially distinct pathways between neurodevelopmental disorders and physiological changes induced by food-related stress.

## 4. Discussion

This manuscript highlights the sex-specific impact of prenatal adversity/stressors such as alcohol and food-related stress on epigenetic patterns of the prefrontal cortex. We show that PAE can cause both sex-concordant and sex-specific changes to DNAm levels, which may explain some of the sexually dimorphic effects of PAE and phenotypic overlaps with neurodevelopmental disorders such as ASD. The pair-fed condition, which models food scarcity/insecurity, demonstrates that exposure to the maternal stress of hunger and disrupted feeding schedules can also alter DNAm patterns of the PFC, which may have long-term consequences on brain function and downstream neurobiological, physiological, and behavioral processes.

### 4.1. PAE-Specific Alterations

Utilizing this same rat model of prenatal alcohol exposure, we have previously shown that PAE can alter the DNAm profile of the hypothalamus and white blood cells in females [53]. Perhaps not surprisingly, none of the PAE-specific DMRs identified in our previous study overlapped with those identified in the present study on the PFC, despite using animals from the same set of litters at the same age. However, we found similar alterations to biological processes involved in immune function and cellular metabolism, suggesting that common pathways are indeed influenced across tissues and brain regions, even though tissue- and brain region-specific effects exist and specific regions of the epigenome may vary.

We further extended our previous work by examining the sex-concordant and sex-specific alterations to DNAm patterns induced by PAE. Similar to most epigenetic studies of PAE, we observed a general hypomethylation in response to PAE, likely as a result of the effects of alcohol on one-carbon metabolism during development [33]. We note that these effects were present across all analyses, suggesting that they are not sex-specific. These results are in line with a study of DNAm and choline supplementation in PAE animals, which showed no significant differences between sexes [72], suggesting that interventions to rescue PAE effects on one-carbon metabolism [73,74] may be effective in both males and females, without the need for sex-specific approaches. Similarly, the majority of DMRs in the PFC were linked to sex-concordant alterations, with a large proportion falling within potassium channel and ion signaling genes, which are closely linked to brain disorders [75]. Of note, potassium channels have recently been proposed as therapeutic targets for epilepsy and intellectual disability [76], which are common co-morbidities of FASD [51]. As such, it is tempting to speculate that the high proportion of these genes in sex-concordant DMRs may point to an underlying mechanism driving some of the phenotypic outcomes of PAE in human populations.

Beyond the broad and sex-concordant effects of PAE on DNAm, it is also possible that the sex-specific DMRs reflect some of the sexual dimorphisms observed for the cognitive and behavioral deficits linked to FASD. In particular, several genes that were linked to PAE in either males or females are involved in the regulation of cell adhesion and brain organization [77,78], such as *Cdh13* and *Itgbl1*, as well as genes related to cortical development [79,80], such as *Tead1* and *Erbb4*. These findings may reflect overall sex-dependent structural differences in the PFC of PAE animals; indeed, structural differences between male and female brains have been reported in several brain imaging studies of individuals with FASD. For example, boys with PAE display larger differences in cortical volume than girls compared to their control counterparts across development [81], and sex differences in cortical thickness and brain volume in childhood have also been reported [82]. Adolescents with FASD also show sex-specific differential activation of the frontal, medial, and temporal cortices compared to controls, further suggesting that PAE-induced epigenetic alterations may have important and sex-dependent downstream effects on behavior and cognition [27]. In addition to this potential relationship with structural alterations, one of the female-specific DMRs was located in *Stk3*, a gene previously associated with intellectual disability [83]. By contrast, male-specific DMRs showed alterations to hormonal regulation and metabolic processes, pointing to key differences in the reprogramming of broader physiological and cognitive functions of the PFC between sexes. Taken together, these findings highlight the potential role of epigenetic modifications in the PFC in driving the sex differences identified in individuals with FASD across multiple domains of cognitive, behavioral, physiological, metabolic, and executive function.

### 4.2. Prenatal Food-Related Stress-Induced Alterations

We also identified a unique epigenetic signature of pair-feeding effects in the PFC. As noted, the pair-fed group in the PAE model is the standard control for the effects of alcohol in reducing food intake [84]. However, compared to the PAE group that, albeit eating less, eats ad libitum, pair-feeding is a treatment in itself, with the PF dams receiving a restricted ration, which results in both hunger and a disrupted feeding schedule. These stress-related effects could potentially parallel or model food scarcity or food insecurity in human populations. As such, the altered epigenetic patterns we observed in the PFC may provide insight into possible alterations that could result from such stressors during development in children. Similar to previous studies of famine in humans and food deprivation in animal models, we observed more DMRs that showed decreased DNAm rather than increased DNAm in PF animals, suggesting that food-related stress may also interfere with one-carbon metabolism and the pathways that deposit methylation on DNA. We also identified a sex-concordant DMR that showed decreased DNAm in PF animals in the glucocorticoid receptor *Nr3c1*, which plays a key role in stress responsivity and may reflect a reprogramming of the stress response. This finding is in contrast to previous work from our group that found no differences in the expression of *Nr3c1* in the hippocampus of PF or PAE animals, suggesting that the effects we observed may be brain region specific [33]. However, a previous study of offspring from dams fed an isocaloric protein-deficient diet before pregnancy found a similar decrease in DNAm in *Nr3c1*, suggesting that these effects may be due to the stress of reduced food intake, which results following any nutrient deficiency in the diet, rather than the nutritional deficits previously described [85]. This result is in line with previous studies that have shown that pair-feeding is a considerable stressor on dams, with lasting consequences on the development, behavior, and physiology of their offspring. As such, altered DNAm of this key HPA axis gene may reflect broader alterations to stress response systems, which may in turn, influence the programming of numerous physiological systems linked to the stress response, including immune function, metabolic processes, and circadian rhythms. Indeed, we observed an association between pair feeding and altered DNAm in biological processes related to these same pathways, further suggesting that exposure to chronic food-related stress during development can have widespread effects on physiological processes.

We also found that the effects of prenatal restricted feeding differed between males and females, with potentially long-term consequences on the functioning of biological systems and disease risk. Previous studies of the offspring of women pregnant during the Dutch Hunger Winter also identified sex-specific effects of food insufficiency on DNAm patterns [45], alongside sex-specific alterations to brain size [42], increased risk of affective disorders in males [86], and altered lipid profiles in females [40]. The latter is of particular note, as female-specific DMRs in the present study showed an enrichment for metabolic processes related to lipid biosynthesis. Although the PFC is not primarily involved in lipid metabolism, alterations to epigenetic patterns of the brain may point to a broader physiological response to prenatal food-related stress that influences tissues throughout the body. In contrast to females, males showed an enrichment of processes related to carbohydrate processing, suggesting fundamental differences in the pathways influenced by food-related stress or disordered eating patterns between sexes. As brain activity and cognitive performance are closely tied to metabolism [87], these metabolic alterations may reflect profound changes in PFC function, which may ultimately influence the neurobiological and behavioral effects of prenatal food scarcity and stress.

### 4.3. Common Impacts of Prenatal Stressors

Beyond the specific impacts of PAE and food-related stress, our results point to common effects of prenatal stressors on the epigenomic state of the cells within the brain, which may highlight pathways underlying more general responses to stressors. It is noteworthy that prenatal alcohol exposure and pair-feeding can have overlapping effects on aspects of development. Similar to pair-feeding, PAE results in reduced food intake, which can alter aspects of HPA activity and regulation [88], reproductive development and function [37], development and activity of the immune system [39,89], as well as depressive- and anxiety-like behavior [12,24]. Thus, while the PAE and PF conditions differ in the type of early life challenge they represent, these early life stressors or adversities may target similar aspects of brain and organ development and thus result in parallel outcomes that, in many instances, may be sex-dependent or sexually dimorphic. Importantly, both PAE and pair-feeding can result in HPA dysregulation, albeit possibly through different mechanisms [36], which can have widespread programming effects on both epigenetic and physiological processes during development. As our analyses parsed out the specific effects of PAE and food-related stress, our results likely reflect broader alterations caused by alterations in endocrine and immune pathways during prenatal development.

Previous studies have shown that maternal stress during development can have profound effects on offspring physical and mental health [90], as well as epigenetic processes [91]. Similarly, we found sex-concordant DNAm alterations in several risk genes involved in mental health disorders. For instance, CACNA1C is a gene involved in synaptic plasticity that has been linked to bipolar disorder, schizophrenia, major depressive disorder, and ASD [92]. There is also evidence that CACNA1C interacts with stress to cause depressive symptoms [93], which, combined with evidence of increased depressive-like symptoms in PAE and PF animals, suggests that the DNAm alterations observed following prenatal stress may prime or sensitize the organism, increasing vulnerability to adverse mental health outcomes. In addition to CACNA1C, we found several DMRs in Pcdh9, another susceptibility gene for depression [94], further highlighting that the shared pathways between prenatal stressors may reprogram key biological systems involved in mental health. Finally, we identified a DMR in two genes involved in the dopaminergic system, *Nrg2* and *Drd4*, suggestive of stress-induced alterations to dopamine regulation, with downstream effects on attention and reward pathways. Importantly, *Drd4* was previously linked to PAE in a study of DNAm in the rat hypothalamus [53], as well as three prior studies of FASD in humans [62,63,64]. This finding suggests that the shared effects of prenatal stressors may vary based on the brain region or tissue examined. These results also emphasize the inexorable link between PAE and early (both pre- and postnatal) life adversity, experienced disproportionately by individuals with FASD, and that cannot be fully disentangled [10]. However, these findings also point to potential genes that can be targeted for therapeutic interventions to reduce the overall impact of prenatal stressors on well-being and risk for disease.

### 4.4. Overlaps between Prenatal Stressors and Autism Spectrum Disorder

Despite differences in the core phenotypic characteristics of FASD and ASD, these neurodevelopmental disorders share several phenotypic characteristics [50], which include deficits in social and communicative functioning [95], socially inappropriate behaviors and difficulty with peers [50], as well as hyperactivity, impulsivity, emotional lability, and difficulty changing strategies or inflexibility [96]. Moreover, co-morbidity between PAE/FASD and ASD or autism-like symptoms has been reported by several groups [51,52]. Case reports on children from the toddler years up to 15 years of age [96,97] were among the first publications to provide data on comorbidity, identifying behavioral alterations characteristic of ASD in children diagnosed with FASD, such as: impaired social behavior, peer relationships and social reciprocity; delays or deficits in verbal and nonverbal communication; lack of make believe and social imitative play; restricted repertories of activities and interests; resistance to change; tactile defensiveness/abnormal sensory responses; and stereotyped motor behaviors. Studies on larger cohorts of individuals with FASD, ASD, and other neurodevelopmental disorders also support an association between heavy PAE and ASD. For example, exploratory data from a diagnostic clinic found that of 21 individuals with FASD, 16 (72%) met ICD-10 criteria for childhood autism [98]. Together, these findings underscore the fact that ASD can be comorbid with FASD and suggest that some common pathways may underlie ASD and FASD. The fact that these comorbidities are not widely recognized may suggest that for some individuals, a diagnosis of FASD precludes secondary diagnoses, such as autism, and conversely, children diagnosed with ASD may not be investigated for possible FASD [97]. This points to the need for more comprehensive approaches to diagnosis for both FASD and ASD.

Importantly, our current and prior findings suggest a link between the epigenomic mechanisms that may underlie these disorders. In our recent epigenome-wide study of individuals with FASD, we found an enrichment of ASD-related genes in these individuals [63], highlighting a potential link between FASD and ASD phenotypes and underlying biological pathways. Similarly, we observed some overlapping genes between FASD and ASD in the present study. Of note, one of the PAE DMRs overlapped with one of the strongest genetic signals of ASD in human populations, NEGR1, which was one of four replicated genes in the largest genome-wide association study to date (*N* = 18,381 ASD cases) [65]. This gene is an adhesion protein that modulates synapse formation and plasticity in the hippocampus and cortex [99]. Importantly, NEGR1 has also been linked to other psychiatric disorders, such as schizophrenia, depression, and Alzheimer’s disease, as well as human intelligence and dyslexia. Collectively, these findings point to a potential role for NEGR1 in neural function and mental health disorders and highlight some of the overlapping phenotypes and deficits present in individuals with ASD and FASD. However, these results should be somewhat tempered by the fact that genetic and epigenetic variation are distinct and further investigation is required to determine whether they have similar effects on gene expression and downstream phenotypes.

Additional overlaps of DNAm profiles in PAE with those in the brains of individuals with ASD were also observed, in particular, several genes linked to DMRs shared between the PAE and PF groups, further emphasizing potential common pathways between ASD, FASD, and developmental outcomes linked to prenatal stressors. For instance, we identified common alterations to *Nrg2*, a gene involved in the dopaminergic system, which is dysregulated in FASD [17], and may highlight common etiologies between prenatal stressors and ASD. By contrast, we found no overlaps with DMRs associated with pair-feeding or food-related stress alone. Although there were fewer genes in this subset, this result may point to a more general role for stress in the common pathways to neurodevelopmental outcomes, with fewer effects observed when narrowing in on specific subtypes of stress, such as in the case of food-related stress. Perhaps most surprisingly, almost every gene overlapping with those in studies of ASD was linked to sex-concordant alterations in the PFC, despite ASD primarily affecting males. This finding suggests that the pathways underlying the overlapping phenotypes between ASD and those resulting from prenatal stressors may be agnostic to the effects of sex, though they may manifest through varying phenotypes between males and females.

### 4.5. Limitations

Our study had some limitations. First, due to the nature of meDIP-seq, we could not quantitatively assess the change in DNAm between exposure groups. Instead, we observed changes in enrichment patterns of DMRs; nonetheless these findings provide insight into larger-scale alterations to the DNA methylome as a result of prenatal exposures. A related point is that we did not assess changes to 5-hydroxymethylcytosine (5hmC), as the antibody used in the meDIP-seq procedure was specific to 5-methylcytosine. However, 5hmC is prevalent in neural tissues and represents an important area of future investigation for epigenome-wide studies [100]. Second, we cannot rule out that some of the observed differences were due to changes in cell type composition resulting from prenatal adversity/stress or inherent to each sex. Although we narrowed our focus to a specific brain region, as opposed to the entire brain, to limit such effects, future studies could aim to measure epigenetic modifications in specific cell types or move toward single-cell analyses to fully uncover the neurobiological mechanisms influenced by PAE and other stressors. Third, although our pair-feeding paradigm models food-related stress and disordered eating in humans, it remains an imperfect measure, which limits these comparisons. Nevertheless, we observed findings similar to those observed in human populations that experience food scarcity, suggesting that we tapped into common biological pathways related to food scarcity or insecurity. More targeted and refined studies are needed to uncover more specifically the biological impacts of food-related stress on the brain. Finally, our sample size was relatively small, limiting our ability to detect sex-specific effects, as exemplified by the lower number of DMRs in the sex-specific analyses. However, a contributing factor may be the age of testing; we examined animals at weaning, which is 22 days of age, well before the onset of puberty, when sex differences begin to fully emerge. As such, subsequent studies should examine epigenetic changes before and after pubertal onset to gain a deeper understanding of PAE-induced sexual dimorphisms. Finally, the functional role of these DNAm alterations remain unknown and should be further investigated. Although DNAm levels are linked to gene expression and downstream cellular functions, the effects of DNAm vary based on its location. For instance, increased DNAm in promoters is linked to reduced gene expression, while the converse is true in gene bodies [101]. DNAm levels at specific CpGs are also associated with changes in transcription factor binding affinities, which, in turn, can influence the expression levels of certain genes [102]. Given these limitations, future studies should assess which specific sites underlie the observed differences in DNAm enrichment and determine whether these DNAm differences lead to changes in gene expression and/or downstream protein levels. Together, these insights would provide a deeper understanding of the cellular and physiological consequences of prenatal stressors on the PFC.

## 5. Conclusions

This study highlights the complex network of neurobiological pathways that respond to prenatal adversity/stressors and that modulate the differential effects of early life insults on functional and health outcomes. Our results also point to some key genes that may drive the phenotypic and biological overlaps between FASD and ASD, pinpointing genes that may influence the manifestation of symptoms or phenotypes present in both disorders. Identifying common neurobiological pathways may provide insight into the biological underpinnings common to FASD and ASD, as well as the downstream consequences of prenatal adversity or stress. Finally, the study of these exposures provides a unique opportunity to investigate the sex-specific effects of prenatal adversity on epigenetic patterns, as the possible biological mechanisms underlying sex-specific responses to prenatal insults are understudied and remain largely unknown. Taken together, the insights provided by our data may ultimately help to identify novel therapeutic targets for the prevention of the adverse consequences of prenatal adversity and the treatment of neurodevelopmental disorders.

## Figures and Tables

**Table 1:** Overlap of genes linked to autism spectrum disorder with differentially methylated regions.

Study	Sample	Age at Collection	Tissue	Overlapping Genes		
				PAE	PF	Shared
Genome-wide association study (GWAS)						
Grove 2019 [65]	18,381 ASD cases 27,969 controls			NEGR1*		MMS22L
Epigenome-wide association studies (EWAS)						
Andrews 2018 [67]	796 ASD cases 858 controls	4–18 years	Blood			
Berko 2014 [68]	47 ASD cases 48 controls	2–17 years	Buccal epithelial cells			NRG2^+^
Hannon 2018 [66]	629 ASD cases 634 controls	Birth	Neonatal blood spots			
Ladd Acosta 2014 [71]	19 ASD cases 21 controls	2–51 years	Prefrontal cortex; Temporal cortex; Cerebellum			
Nardone 2017 [69]	16 male ASD cases 15 male controls	17–68 years	Frontal cortex			NEDD4L
Wong 2019 [70]	36 ASD cases 33 controls	29.3 years (±28.2)	Prefrontal cortex	CDH13^+^		PRKAR1B
	33 ASD cases 38 controls	29.1 years (±18.6)	Temporal cortex			
	34 ASD cases 29 controls	30.6 years (±21.2)	Cerebellum			
	30 ASD cases 29 controls	29.0 years (±18.9)	Prefrontal and temporal cortex, analyzed together	GRIK1		FRMD4A

ASD = autism spectrum disorder; PAE = prenatal alcohol exposed; PF = pair-fed (exposed to food-related stress); shared = DMRs shared between PAE and PF compared to controls. *NEGR1 was one of four genes replicated in an independent sample by Grove et al. (2019) [65] and is one of the strongest loci for ASD. ^+^All overlapping genes were linked to sex-concordant differentially methylated regions (DMRs), with the exception of CDH13, which had both sex-concordant and male-specific DMRs in response to PAE, and NRG2, which had a female-specific DMR.

## Data Availability

Data have been uploaded to the Gene Expression Omnibus (GEO) under accession number GSE186840. Original code can be found at github.com/alussier17/alussier_scripts/ (accessed on 8 November 2021).

## Supplementary Materials

The following are available online at https://www.mdpi.com/article/10.3390/genes12111773/s1, Table S1: PAE-specific DMRs, Table S2: PAE-specific gene ontology, Table S3: PF-specific DMRs, Table S4: PF-specific gene ontology, Table S5: PAE+PF shared DMRs, Table S6: PAE+PF shared gene ontology.

## References

[1] Ronald A., Pennell C.E., Whitehouse A.J. (2011). Prenatal Maternal Stress Associated with ADHD and Autistic Traits in Early Childhood. Front. Psychol..

[2] Beversdorf D.Q., Stevens H.E., Margolis K.G., Van de Water J. (2019). Prenatal Stress and Maternal Immune Dysregulation in Autism Spectrum Disorders: Potential Points for Intervention. Curr. Pharm. Des..

[3] Andalib S., Emamhadi M.R., Yousefzadeh-Chabok S., Shakouri S.K., Høilund-Carlsen P.F., Vafaee M.S., Michel T.M. (2017). Maternal SSRI Exposure Increases the Risk of Autistic Offspring: A Meta-Analysis and Systematic Review. Eur. Psychiatry.

[4] Karimi P., Kamali E., Mousavi S.M., Karahmadi M. (2017). Environmental Factors Influencing the Risk of Autism. J. Res. Med. Sci..

[5] Bird A. (2007). Perceptions of Epigenetics. Nature.

[6] Aristizabal M.J., Anreiter I., Halldorsdottir T., Odgers C.L., McDade T.W., Goldenberg A., Mostafavi S., Kobor M.S., Binder E.B., Sokolowski M.B. (2020). Biological Embedding of Experience: A Primer on Epigenetics. Proc. Natl. Acad. Sci. USA.

[7] Boyce W.T., Kobor M.S. (2015). Development and the Epigenome: The ‘Synapse’ of Gene-Environment Interplay. Dev. Sci..

[8] Bertrand J., Floyd L.L., Weber M.K. (2005). Fetal Alcohol Syndrome Prevention Team, Division of Birth Defects and Developmental Disabilities, National Center on Birth Defects and Developmental Disabilities, Centers for Disease Control and Prevention (CDC). Guidelines for identifying and referring persons with fetal alcohol syndrome. MMWR Recomm. Rep..

[9] Weinberg J., Sliwowska J.H., Lan N., Hellemans K.G.C. (2008). Prenatal Alcohol Exposure: Foetal Programming, the Hypothalamic-Pituitary-Adrenal Axis and Sex Differences in Outcome. J. Neuroendocrinol..

[10] Kaitlyn M., Rasmussen C., Oberlander T.F., Loock C., Pei J., Andrew G., Reynolds J., Weinberg J. (2016). Dysregulation of the Cortisol Diurnal Rhythm Following Prenatal Alcohol Exposure and Early Life Adversity. Alcohol.

[11] Kable A.J., Mehta P.K., Coles C.D. (2021). Alterations in Insulin Levels in Adults with Prenatal Alcohol Exposure. Alcohol. Clin. Exp. Res..

[12] Hellemans K.G., Sliwowska J.H., Verma P., Weinberg J. (2010). Prenatal Alcohol Exposure: Fetal Programming and Later Life Vulnerability to Stress, Depression and Anxiety Disorders. Neurosci. Biobehav. Rev..

[13] Bodnar T.S., Weinberg J. (2013). Prenatal Alcohol Exposure: Impact on Neuroendocrine—Neuroimmune Networks.

[14] Glover V. (2014). Maternal Depression, Anxiety and Stress During Pregnancy and Child Outcome; What Needs to Be Done. Best Pract. Res. Clin. Obstet. Gynaecol..

[15] Van den Bergh B.R.H., van den Heuvel M.I., Lahti M., Braeken M., de Rooij S.R., Entringer S., Hoyer D., Roseboom T., Räikkönen K., King S. (2020). Prenatal Developmental Origins of Behavior and Mental Health: The Influence of Maternal Stress in Pregnancy. Neurosci. Biobehav. Rev..

[16] Ruggiero M.J., Boschen K.E., Roth T.L., Klintsova A.Y. (2018). Sex Differences in Early Postnatal Microglial Colonization of the Developing Rat Hippocampus Following a Single-Day Alcohol Exposure. J. Neuroimmune Pharmacol..

[17] Uban K.A., Comeau W.L., Ellis L.A., Galea L.A., Weinberg J. (2013). Basal Regulation of HPA and Dopamine Systems Is Altered Differentially in Males and Females by Prenatal Alcohol Exposure and Chronic Variable Stress. Psychoneuroendocrinology.

[18] Lee S., Rivier C. (1996). Gender Differences in the Effect of Prenatal Alcohol Exposure on the Hypothalamic-Pituitary-Adrenal Axis Response to Immune Signals. Psychoneuroendocrinology.

[19] Kelly S.J., Day N., Streissguth A.P. (2000). Effects of Prenatal Alcohol Exposure on Social Behavior in Humans and Other Species. Neurotoxicology Teratol..

[20] Holman P.J., Baglot S.L., Morgan E., Weinberg J. (2019). Effects of Prenatal Alcohol Exposure on Social Competence: Asymmetry in Play Partner Preference among Heterogeneous Triads of Male and Female Rats. Dev. Psychobiol..

[21] Holman P.J., Raineki C., Chao A., Grewal R., Haghighat S., Fung C., Morgan E., Ellis L., Yu W., Weinberg J. (2021). Altered Social Recognition Memory and Hypothalamic Neuropeptide Expression in Adolescent Male and Female Rats Following Prenatal Alcohol Exposure and/or Early-Life Adversity. Psychoneuroendocrinology.

[22] Caldwell K.K., Sheema S., Paz R.D., Samudio-Ruiz S.L., Laughlin M.H., Spence N.E., Roehlk M.J., Alcon S.N., Allan A.M. (2008). Fetal Alcohol Spectrum Disorder-Associated Depression: Evidence for Reductions in the Levels of Brain-Derived Neurotrophic Factor in a Mouse Model. Pharmacol. Biochem. Behav..

[23] Raineki C., Hellemans K.G.C., Bodnar T., Lavigne K.M., Ellis L., Woodward T.S., Weinberg J. (2014). Neurocircuitry Underlying Stress and Emotional Regulation in Animals Prenatally Exposed to Alcohol and Subjected to Chronic Mild Stress in Adulthood. Front. Endocrinol..

[24] Lam V.Y.Y., Raineki C., Ellis L., Yu W., Weinberg J. (2018). Interactive Effects of Prenatal Alcohol Exposure and Chronic Stress in Adulthood on Anxiety-Like Behavior and Central Stress-Related Receptor mRNA Expression: Sex- and Time-Dependent Effects. Psychoneuroendocrinology.

[25] Thanh N.X., Jonsson E., Salmon A., Sebastianski M. (2014). Incidence and Prevalence of Fetal Alcohol Spectrum Disorder by Sex and Age Group in Alberta, Canada. J. Popul. Ther. Clin. Pharmacol..

[26] Woods K.J., Thomas K.G.F., Molteno C.D., Jacobson J.L., Jacobson S.W., Meintjes E.M. (2018). Prenatal Alcohol Exposure Affects Brain Function During Place Learning in a Virtual Environment Differently in Boys and Girls. Brain Behav..

[27] Tesche C.D., Kodituwakku P.W., Garcia C.M., Houck J.M. (2015). Sex-Related Differences in Auditory Processing in Adolescents with Fetal Alcohol Spectrum Disorder: A Magnetoencephalographic Study. NeuroImage.

[28] Uban K.A., Herting M.M., Wozniak J.R., Sowell E.R. (2017). Sex Differences in Associations between White Matter Microstructure and Gonadal Hormones in Children and Adolescents with Prenatal Alcohol Exposure. Psychoneuroendocrinology.

[29] Sayal K., Heron J., Golding J., Emond A. (2007). Prenatal Alcohol Exposure and Gender Differences in Childhood Mental Health Problems: A Longitudinal Population-Based Study. Pediatrics.

[30] Lussier A.A., Weinberg J., Kobor M.S. (2017). Epigenetics Studies of Fetal Alcohol Spectrum Disorder: Where Are We Now?. Epigenomics.

[31] Petrelli B., Weinberg J., Hicks G.G. (2018). Effects of Prenatal Alcohol Exposure (PAE): Insights into FASD Using Mouse Models of PAE. Biochem. Cell Biol..

[32] Schaffner S.L., Lussier A.A., Baker J.A., Goldowitz D., Hamre K.M., Kobor M.S. (2020). Neonatal Alcohol Exposure in Mice Induces Select Differentiation- and Apoptosis-Related Chromatin Changes Both Independent of and Dependent on Sex. Front. Genet..

[33] Ngai Y.F., Sulistyoningrum D.C., O’Neill R., Innis S.M., Weinberg J., Devlin A.M. (2015). Prenatal Alcohol Exposure Alters Methyl Metabolism and Programs Serotonin Transporter and Glucocorticoid Receptor Expression in Brain. Am. J. Physiology Regul. Integr. Comp. Physiol..

[34] Gatev E., Inkster A.M., Negri G.L., Konwar C., Lussier A.A., Skakkebaek A., Sokolowski M.B., Gravholt C.H., Dunn E.C., Kobor M.S. (2021). Autosomal Sex-Associated Co-Methylated Regions Predict Biological Sex from DNA Methylation. Nucleic Acids Res..

[35] Glavas M.M., Ellis L., Yu W.K., Weinberg J. (2007). Effects of Prenatal Ethanol Exposure on Basal Limbic-Hypothalamic-Pituitary-Adrenal Regulation: Role of Corticosterone. Alcohol. Clin. Exp. Res..

[36] Lan N., Chiu M.P.Y., Ellis L., Weinberg J. (2017). Prenatal Alcohol Exposure and Prenatal Stress Differentially Alter Glucocorticoid Signaling in the Placenta and Fetal Brain. Neuroscience.

[37] Sliwowska J.H., Comeau W.L., Bodnar T.S., Ellis L., Weinberg J. (2016). Prenatal Alcohol Exposure and Pair Feeding Differentially Impact Puberty and Reproductive Development in Female Rats: Role of the Kisspeptin System. Alcohol. Clin. Exp. Res..

[38] Gawałek M., Sliwowska J.H. (2015). Neuronal Basis of Reproductive Dysfunctions Associated with Diet and Alcohol: From the Womb to Adulthood. Reprod. Biol..

[39] Bodnar T.S., Hill L.A., Weinberg J. (2016). Evidence for an Immune Signature of Prenatal Alcohol Exposure in Female Rats. Brain Behav. Immun..

[40] Lumey L.H., Stein A.D., Kahn H.S., Romijn J.A. (2009). Lipid Profiles in Middle-Aged Men and Women after Famine Exposure During Gestation: The Dutch Hunger Winter Families Study. Am. J. Clin. Nutr..

[41] Dearden L., Bouret S.G., Susan E. (2018). Ozanne. Sex and Gender Differences in Developmental Programming of Metabolism. Mol. Metab..

[42] de Rooij S.R., Caan M.W.A., Swaab D.F., Nederveen A.J., Majoie C.B., Schwab M., Painter R.C., Roseboom T.J. (2016). Prenatal Famine Exposure Has Sex-Specific Effects on Brain Size. Brain.

[43] Tobi E.W., Goeman J.J., Monajemi R., Gu H., Putter H., Zhang Y., Slieker R.C., Stok A.P., Thijssen P.E., Müller F. (2014). DNA Methylation Signatures Link Prenatal Famine Exposure to Growth and Metabolism. Nat. Commun..

[44] Heijmans B.T., Tobi E.W., Stein A.D., Putter H., Blauw G.J., Susser E.S., Slagboom P.E., Lumey L.H. (2008). Persistent Epigenetic Differences Associated with Prenatal Exposure to Famine in Humans. Proc. Natl. Acad. Sci. USA.

[45] Tobi E.W., Lumey L.H., Talens R.P., Kremer D., Putter H., Stein A.D., Slagboom P.E., Heijmans B.T. (2009). DNA Methylation Differences after Exposure to Prenatal Famine are Common and Timing- and Sex-Specific. Hum. Mol. Genet..

[46] Sullivan R.M., Gratton A. (2002). Prefrontal Cortical Regulation of Hypothalamic-Pituitary-Adrenal Function in the Rat and Implications for Psychopathology: Side Matters. Psychoneuroendocrinology.

[47] Damasio A.R., Eighth C.U. (2000). Ariens Kappers Lecture. The fabric of the mind: A neurobiological perspective. Prog. Brain Res..

[48] McKlveen J.M., Myers B., Flak J.N., Bundzikova J., Solomon M.B., Seroogy K.B., Herman J.P. (2013). Role of Prefrontal Cortex Glucocorticoid Receptors in Stress and Emotion. Biol. Psychiatry.

[49] Cerqueira J.J., Almeida O.F., Sousa N. (2008). The stressed prefrontal cortex. Left? Right!. Brain Behav. Immun..

[50] Bishop S., Gahagan S., Lord C. (2007). Re-Examining the Core Features of Autism: A Comparison of Autism Spectrum Disorder and Fetal Alcohol Spectrum Disorder. J. Child Psychol. Psychiatry Allied Discip..

[51] Popova S., Lange S., Shield K., Mihic A., Chudley A.E., Mukherjee R.A.S., Bekmuradov D., Rehm J. (2016). Comorbidity of Fetal Alcohol Spectrum Disorder: A Systematic Review and Meta-Analysis. Lancet.

[52] Lange S., Rehm J., Anagnostou E., Popova S. (2018). Prevalence of Externalizing Disorders and Autism Spectrum Disorders among Children with Fetal Alcohol Spectrum Disorder: Systematic Review and Meta-Analysis. Biochem. Cell Biol..

[53] Lussier A.A., Bodnar T.S., Mingay M., Morin A.M., Hirst M., Kobor M.S., Weinberg J. (2018). Prenatal Alcohol Exposure: Profiling Developmental DNA Methylation Patterns in Central and Peripheral Tissues. Front. Genet..

[54] Taiwo O., Wilson G., Morris T., Seisenberger S., Reik W., Pearce D., Beck S., Butcher L. (2012). Methylome Analysis Using MeDIP-Seq with Low DNA Concentrations. Nat. Protoc..

[55] Li H., Durbin R. (2009). Fast and Accurate Short Read Alignment with Burrows-Wheeler Transform. Bioinformatics.

[56] Zhang Y., Liu T., Meyer C., Eeckhoute J., Johnson D.S., Bernstein B.E., Nusbaum C., Myers R.M., Brown M., Li W. (2008). Model-Based Analysis of ChIP-Seq (MACS). Genome Biol..

[57] Stark R., Brown G. (2011). Diffbind: Differential Binding Analysis of ChIP-Seq Peak Data. Bioconductor.

[58] Ross-Innes C.S., Stark R., Teschendorff A.E., Holmes K.A., Ali H.R., Dunning M.J., Brown G.D., Gojis O., Ellis I.O., Green A.R. (2012). Differential Oestrogen Receptor Binding Is Associated with Clinical Outcome in Breast Cancer. Nature.

[59] Benjamini Y., Hochberg Y. (1995). Controlling the False Discovery Rate: A Practical and Powerful Approach to Multiple Testing. J. R. Stat. Soc. Ser. B.

[60] Gillis J., Mistry M., Pavlidis P. (2010). Gene function analysis in complex data sets using ErmineJ. Nature Protocols.

[61] Lee H.K., Braynen W., Keshav K., Pavlidis P. (2005). ErmineJ: Tool for Functional Analysis of Gene Expression Data Sets. BMC Bioinform..

[62] Lussier A.A., Morin A.M., MacIsaac J.L., Salmon J., Weinberg J., Reynolds J.N., Pavlidis P., Chudley A.E., Kobor M.S. (2018). DNA Methylation as a Predictor of Fetal Alcohol Spectrum Disorder. Clin. Epigenetics.

[63] Portales-Casamar E., Lussier A.A., Jones M.J., MacIsaac J.L., Edgar R.D., Mah S.M., Barhdadi A., Provost S., Lemieux-Perreault L., Cynader M.S. (2016). DNA Methylation Signature of Human Fetal Alcohol Spectrum Disorder. Epigenetics Chromatin.

[64] Fransquet P.D., Hutchinson D., Olsson C.A., Wilson J., Allsop S., Najman J., Elliott E., Mattick R.P., Saffery R., Ryan J. (2016). Perinatal Maternal Alcohol Consumption and Methylation of the Dopamine Receptor DRD4 in the Offspring: The Triple B Study. Environ. Epigenetics.

[65] Grove J., Ripke S., Als T.D., Mattheisen M., Walters R.K., Won H., Pallesen J., Agerbo E., Andreassen O.A., Anney R. (2019). Identification of Common Genetic Risk Variants for Autism Spectrum Disorder. Nat. Genet..

[66] Hannon E., Schendel D., Ladd-Acosta C., Grove J., Agerbo E., Als T.D., Belliveau R., Bybjerg-Grauholm J., Bækved-Hansen M., Børglum A. (2018). Elevated Polygenic Burden for Autism Is Associated with Differential DNA Methylation at Birth. Genome Med..

[67] Andrews S.V., Sheppard B., Windham G.C., Schieve L.A., Schendel D.E., Croen L.A., Chopra P., Alisch R.S., Newschaffer C.J., Warren S.T. (2018). Case-Control Meta-Analysis of Blood DNA Methylation and Autism Spectrum Disorder. Mol. Autism.

[68] Berko E.R., Suzuki M., Beren F., Lemetre C., Alaimo C.M., Calder R.B., Ballaban-Gil K., Gounder B., Kampf K., Kirschen J. (2014). Mosaic Epigenetic Dysregulation of Ectodermal Cells in Autism Spectrum Disorder. PLoS Genet..

[69] Nardone S., Sams D.S., Zito A., Reuveni E., Elliott E. (2017). Dysregulation of Cortical Neuron DNA Methylation Profile in Autism Spectrum Disorder. Cereb. Cortex.

[70] Wong C.C.Y., Smith R.G., Hannon E., Ramaswami G., Parikshak N.N., Assary E., Troakes C., Poschmann J., Schalkwyk L.C., Sun W. (2019). Genome-Wide DNA Methylation Profiling Identifies Convergent Molecular Signatures Associated with Idiopathic and Syndromic Autism in Post-Mortem Human Brain Tissue. Hum. Mol. Genet..

[71] Ladd-Acosta C., Hansen K.D., Briem E., Fallin M.D., Kaufmann W.E., Feinberg A.P. (2014). Common DNA Methylation Alterations in Multiple Brain Regions in Autism. Mol. Psychiatry.

[72] Otero N.K.H., Thomas J.D., Saski C.A., Xia X., Kelly S.J. (2012). Choline Supplementation and DNA Methylation in the Hippocampus and Prefrontal Cortex of Rats Exposed to Alcohol during Development. Alcohol. Clin. Exp. Res..

[73] Wozniak J.R., Fink B.A., Fuglestad A.J., Eckerle J.K., Boys C.J., Sandness K.E., Radke J.P., Miller N.C., Lindgren C., Brearley A.M. (2020). Four-Year Follow-up of a Randomized Controlled Trial of Choline for Neurodevelopment in Fetal Alcohol Spectrum Disorder. J. Neurodev. Disord..

[74] Wozniak J.R., Fuglestad A.J., Eckerle J.K., Fink B.A., Hoecker H.L., Boys C.J., Radke J.P., Kroupina M.G., Miller N.C., Brearley A.M. (2015). Choline Supplementation in Children with Fetal Alcohol Spectrum Disorders: A Randomized, Double-Blind, Placebo-Controlled Trial. Am. J. Clin. Nutr..

[75] Kumar P., Kumar D., Jha S.K., Jha N.K., Ambasta R.K. (2016). Chapter three—Ion channels in neurological disorders. Adv. Protein Chem. Struct. Biol..

[76] Noh W., Pak S., Choi G., Yang S., Yang S. (2019). Transient Potassium Channels: Therapeutic Targets for Brain Disorders. Front. Cell. Neurosci..

[77] Hirano S., Takeichi M. (2012). Cadherins in Brain Morphogenesis and Wiring. Physiol. Rev..

[78] Schmid R.S., Anton E.S. (2003). Role of Integrins in the Development of the Cerebral Cortex. Cereb. Cortex.

[79] Mukhtar T., Breda J., Grison A., Karimaddini Z., Grobecker P., Iber D., Beisel C., van Nimwegen E., Taylor V. (2020). Tead Transcription Factors Differentially Regulate Cortical Development. Sci. Rep..

[80] Wang H., Liu F., Chen W., Sun X., Cui W., Dong Z., Zhao K., Zhang H., Li H., Xing G. (2018). Genetic Recovery of ErbB4 in Adulthood Partially Restores Brain Functions in Null Mice. Proc. Natl. Acad. Sci. USA.

[81] Lebel C., Mattson S.N., Riley E.P., Jones K.L., Adnams C.M., May P.A., Bookheimer S.Y., O’Connor M.J., Narr K.L., Kan E. (2012). A Longitudinal Study of the Long-Term Consequences of Drinking During Pregnancy: Heavy in Utero Alcohol Exposure Disrupts the Normal Processes of Brain Development. J. Neurosci..

[82] Treit S., Chen Z., Zhou D., Baugh L., Rasmussen C., Andrew G., Pei J., Beaulieu C. (2017). Sexual Dimorphism of Volume Reduction but Not Cognitive Deficit in Fetal Alcohol Spectrum Disorders: A Combined Diffusion Tensor Imaging, Cortical Thickness and Brain Volume Study. NeuroImage.

[83] Gambin T., Yuan B., Bi W., Liu P., Rosenfeld J.A., Coban-Akdemir Z., Pursley A.N., Nagamani S.C.S., Marom R., Golla S. (2017). Identification of Novel Candidate Disease Genes from De Novo Exonic Copy Number Variants. Genome Med..

[84] Weinberg J. (1984). Nutritional Issues in Perinatal Alcohol Exposure. Neurobehav. Toxicol. Teratol..

[85] Burdge G.C., Slater-Jefferies J., Torrens C., Phillips E.S., Hanson M.A., Lillycrop K.A. (2007). Dietary Protein Restriction of Pregnant Rats in the F0 Generation Induces Altered Methylation of Hepatic Gene Promoters in the Adult Male Offspring in the F1 and F2 Generations. Br. J. Nutr..

[86] Brown A.S., Susser E.S., Lin S.P., Neugebauer R., Gorman J.M. (1995). Increased Risk of Affective Disorders in Males after Second Trimester Prenatal Exposure to the Dutch Hunger Winter of 1944–45. Br. J. Psychiatry.

[87] Dienel G.A. (2019). Brain Glucose Metabolism: Integration of Energetics with Function. Physiol. Rev..

[88] Weinberg J. (1985). Effects of Ethanol and Maternal Nutritional Status on Fetal Development. Alcohol. Clin. Exp. Res..

[89] Bodnar T.S., Raineki C., Wertelecki W., Yevtushok L., Plotka L., Zymak-Zakutnya N., Honerkamp-Smith G., Wells A., Rolland M., Woodward T.S. (2018). Altered Maternal Immune Networks Are Associated with Adverse Child Neurodevelopment: Impact of Alcohol Consumption During Pregnancy. Brain Behav. Immun..

[90] Kinsella M.T., Monk C. (2009). Impact of Maternal Stress, Depression and Anxiety on Fetal Neurobehavioral Development. Clin. Obstet. Gynecol..

[91] Cao-Lei L., Laplante D.P., King S. (2016). Prenatal Maternal Stress and Epigenetics: Review of the Human Research. Curr. Mol. Biol. Rep..

[92] Moon A.L., Haan N., Wilkinson L.S., Thomas K.L., Hall J. (2018). CACNA1C: Association with Psychiatric Disorders, Behavior, and Neurogenesis. Schizophr. Bull..

[93] Dedic N., Pöhlmann M.L., Richter J.S., Mehta D., Czamara D., Metzger M.W., Dine J., Bedenk B.T., Hartmann J., Wagner K.V. (2018). Cross-Disorder Risk Gene CACNA1C Differentially Modulates Susceptibility to Psychiatric Disorders During Development and Adulthood. Mol. Psychiatry.

[94] Xiao X., Zheng F., Chang H., Ma Y., Yao Y., Luo X., Li M. (2018). The Gene Encoding Protocadherin 9 (PCDH9), a Novel Risk Factor for Major Depressive Disorder. Neuropsychopharmacology.

[95] Stevens S.A., Nash K., Koren G., Rovet J. (2013). Autism Characteristics in Children with Fetal Alcohol Spectrum Disorders. Child Neuropsychol..

[96] Harris S.R., MacKay L.L., Osborn J.A. (1995). Autistic Behaviors in Offspring of Mothers Abusing Alcohol and Other Drugs: A Series of Case Reports. Alcohol. Clin. Exp. Res..

[97] Nanson J.L. (1992). Autism in Fetal Alcohol Syndrome: A Report of Six Cases. Alcohol. Clin. Exp. Res..

[98] Mukherjee R., Layton M., Yacoub E., Turk J. (2011). Autism and Autistic Traits in People Exposed to Heavy Prenatal Alcohol: Data from a Clinical Series of 21 Individuals and Nested Case Control Study. Adv. Ment. Health Intellect. Disabil..

[99] Singh K., Jayaram M., Kaare M., Leidmaa E., Jagomäe T., Heinla I., Hickey M.A., Kaasik A., Schäfer M.K., Innos J. (2019). Neural Cell Adhesion Molecule Negr1 Deficiency in Mouse Results in Structural Brain Endophenotypes and Behavioral Deviations Related to Psychiatric Disorders. Sci. Rep..

[100] Lister R., Mukamel E.A., Nery J.R., Urich M., Puddifoot C.A., Johnson N.D., Lucero J., Huang Y., Dwork A.J., Schultz M.D. (2013). Global Epigenomic Reconfiguration During Mammalian Brain Development. Science.

[101] Greenberg M.V., Bourc’his D. (2019). The diverse roles of DNA methylation in mammalian development and disease. Nat. Rev. Mol. Cell Biol..

[102] Yin Y., Morgunova E., Jolma A., Kaasinen E., Sahu B., Khund-Sayeed S., Das P.K., Kivioja T., Dave K., Zhong F. (2017). Impact of cytosine methylation on DNA binding specificities of human transcription factors. Science.

